# The polyploid continuum and the landscape of polyploid genomic variation

**DOI:** 10.1002/ajb2.70121

**Published:** 2025-11-02

**Authors:** Alex D. Twyford, Justin L. Conover, Jeff J. Doyle, Annaliese S. Mason, Douglas E. Soltis, Pamela S. Soltis, Jonathan F. Wendel

**Affiliations:** ^1^ Institute of Ecology and Evolution University of Edinburgh Charlotte Auerbach Road Edinburgh EH9 3FL UK; ^2^ Royal Botanic Garden Edinburgh 20A Inverleith Row Edinburgh EH3 5LR UK; ^3^ Donald Danforth Plant Science Center St. Louis 63132 Missouri USA; ^4^ Ecology and Evolutionary Biology Department University of Arizona Tucson 85718 Arizona USA; ^5^ Molecular and Cellular Biology Department University of Arizona Tucson 85718 Arizona USA; ^6^ School of Integrative Plant Science, Sections of Plant Biology and Plant Breeding & Genetics Cornell University Ithaca 14853 New York USA; ^7^ Plant Breeding Department, Institute of Crop Science and Resource Conservation (INRES) University of Bonn Bonn Germany; ^8^ Department of Biology University of Florida Gainesville 32611 Florida USA; ^9^ Florida Museum of Natural History University of Florida Gainesville 32611 Florida USA; ^10^ Department of Ecology, Evolution, and Organismal Biology Iowa State University Ames 50011 Iowa USA

**Keywords:** allopolyploid, autopolyploid, genomes, homoeolog, polyploidy

## Abstract

Polyploid research has traditionally distinguished between autopolyploids and allopolyploids on the basis of evolutionary origins, modes of inheritance, or chromosomal pairing behavior during meiosis. It has long been recognized, however, that a binary classification does not accurately reflect the complexity and diversity inherent to polyploid organisms, and that these definitions may be inadequate to capture biological diversity. Moreover, inferred conditions at polyploid formation are often obscured by numerous post‐polyploidy genomic processes, necessitating a temporal perspective on the meaning of polyploid terminology. In this review, we explore the concept of the “polyploid continuum” and highlight the temporal biological fluidity between the classically recognized alternative end points of autopolyploidy and allopolyploidy. We consider aspects of the polyploid continuum that might meaningfully be evaluated on the basis of genetic variation, including at the sequence, structural, and functional levels. We discuss the utility of the polyploid continuum concept and how it might be visualized as a multidimensional landscape of polyploid diversity that represents a temporal snapshot at any one time. This perspective may better reveal the genesis of polyploid diversity in its many dimensions and provide a framework for understanding the dynamic evolutionary processes that underpin polyploid variation.

Polyploidy—whole‐genome duplication—has attracted the interest of biologists for decades, as it plays a major role in evolution at every level of biological organization, influencing genes, cells, tissues, species, communities, and ecosystems (Ramsey and Ramsey, 2014; Soltis et al., 2016; Doyle and Coate, 2019; Fox et al., 2020). Polyploidy was first discovered in the early twentieth century, when it was observed that individuals or related species differ in multiples of a basic chromosome number (Lutz, 1907), and that two different sets of chromosomes could be present in one species (Kihara, 1930). Following these initial taxonomic and cytogenetic observations, understanding of the genetics of polyploidy increased as the ability to genotype and sequence DNA fueled the burgeoning fields of genetics and, eventually, genomics, which have revealed polyploidy to be an important, recurrent, and widespread process in genome evolution across diverse taxa (Mable, 2003; Jiao et al., 2011; Li et al., 2021; Barker et al., 2024).

Despite many advances in polyploid research, current thinking remains heavily shaped by the foundational work of Kihara and Ono (1926), Clausen et al., (1945), and Stebbins (1947), who discussed the assignment of polyploids to one of two classes, autopolyploids and allopolyploids. Kihara and Ono (1926) were the first to introduce these terms, defining autopolyploids as individuals possessing multiple chromosome sets derived from a single species, and allopolyploids as hybrids formed between two distinct species, followed by chromosome doubling. Stebbins (1947, 1950) later extended these ideas within an evolutionary framework, with his view of autopolyploidy often grounded in synthetic polyploids—experimental creations derived from somatic doubling under laboratory conditions—rather than from naturally occurring examples. Later, Harlan and de Wet (1975) challenged the “hybridization followed by chromosome doubling” hypothesis for allopolyploid origins, introducing the now well‐established idea that the majority of all polyploids arise sexually, rather than somatically, via hybridization events involving unreduced gametes (gametes with the somatic chromosome number) (reviewed by Ramsey and Schemske, 1998; Mason and Pires, 2015; Kreiner et al., 2017; Clo et al., 2022). This shifted the original definition of autopolyploid to include events that occurred within a species to produce a polyploid (including hybridization between different populations of that species). This definition of polyploidy, based on mode of origin, separates autopolyploids that arise within species from allopolyploids that arise via hybridization between species (Carputo et al., 2006; Lv et al., 2024) and is thus “taxonomic” and not “genetic” (Doyle and Sherman‐Broyles, 2017).

Aside from a definition of polyploids based on mode of origin, polyploid types can be defined by cytological characteristics and meiotic behavior of the chromosomes. Before genotyping and DNA sequencing, chromosome size and appearance were thought to determine whether chromosomes would pair or not, and pairing was subsequently also used to define polyploid types (Kihara, 1930; Stebbins, 1950). Allopolyploids were thought to exhibit strict bivalent pairing, whereas autopolyploids showed frequent multivalents (arising from associations between the multiple copies of each homologous chromosome; Stebbins, 1950). The cases where two sets of chromosomes were clearly present but multivalent pairing was still observed were separated by Stebbins (1947) into a third category of “segmental allopolyploids” representing an intermediate between auto‐ and allopolyploidy. These original cytological definitions did not stand the test of time as our understanding of meiotic mechanisms, DNA sequence similarity, and genomic relationships has improved (reviewed by Soltis et al., 1993; Sybenga, 1996; Ramsey and Schemske, 2002; Mason and Wendel, 2020; Lv et al., 2024): multivalent formation occurs in almost all polyploid taxa at some frequency and is mainly related to genetic control of meiosis (reviewed by Nyarko and Mason, 2022). Later on, the cytological definition took on greater utility, as marker inheritance in segregating progeny revealed that bivalent chromosome pairing had two possible genetic outcomes: disomic inheritance, where each chromosome has a single preferred pairing partner (allopolyploids), and polysomic inheritance (autopolyploids), where multiple homologous copies of each chromosome can pair randomly (Cifuentes et al., 2010; Zielinski and Mittelsten Scheid, 2012; Lv et al., 2024).

It has long been known that the two categories of auto‐ and allopolyploidy, no matter how they are defined, fail to capture the true biological diversity of polyploid formation and outcomes (Stebbins, 1947; Grant, 1981; Jackson, 1982; Soltis et al., 2010), and that classifying polyploids based on different definitions can create conflicting and confusing categorizations. For example, a polyploid that formed through interspecific hybridization and has polysomic inheritance could be considered a taxonomic allopolyploid and a cytological autopolyploid simultaneously (e.g., allopolyploid *Chrysanthemum morifolium*, which has a hybrid origin but shows hexasomic inheritance; Fan et al., 2022; Song et al., 2023). While the end points of polyploid genomic diversity are often distinct in many ways, between these end points is a complex “gray zone” (Doyle and Sherman‐Broyles, 2017) in which polyploids may demonstrate diverse chromosome‐pairing behaviors and inheritance patterns as well as variations in the genomic characters now available to systematists and evolutionary biologists (Meirmans and Van Tienderen, 2013).

Here, we explore the nature of polyploid genomic diversity in light of recent insights from comparative genomics, which have revealed the evolutionary dynamics of polyploid genomes post‐formation. We focus on the possibility that autopolyploidy and allopolyploidy—while conventionally treated as distinct—may be temporally interconnected. We focus most of our initial discussion on “contemporary polyploids”—cases where closely related species differ by (nearly) whole sets of base chromosome numbers—before addressing deeper time, now that we understand that all land plant genomes have a history that includes episodes of ancient polyploidy (Jiao et al., 2011; One Thousand Plant Transcriptomes Initiative, 2019). Our goals are to illuminate the inherently temporal dimension of polyploidy “states” and present a forward‐looking perspective on the future of polyploid research, focusing on what will be achievable on the basis of comparative genomic analyses of complete haplotype‐resolved annotated genomes, which will become increasingly available for non‐model polyploid species. We aim to bring explicit attention to the relative fluidity and diversity of polyploid conditions, highlight the processes at play that underlie variation and transitions across polyploid types in nature, and make testable predictions for future studies. We start by discussing the ways that polyploidy diversity better fits a continuum than a binary model, and the genomic features that may characterize this continuum, before considering the complexities introduced by multiple processes that might generate transitions between genomic features, including *apparent* modes of origin, over time. We conclude by examining how our perception of polyploid variation influences our approach to addressing fundamental questions in polyploid genomic research.

## CLASSIFICATION AND THE POLYPLOID CONTINUUM

There is increasing recognition that polyploids do not fit neatly into categories of autopolyploids and allopolyploids, using any of the traditional definitions of taxonomy/evolutionary origins, modes of inheritance, or chromosomal pairing behavior at meiosis (Box 1). Many others have noted this inadequacy, describing the cytogenetic, genomic, and taxonomic variation present across the diversity of polyploids as being a continuum, potentially along several axes of variation (Wendel and Doyle, 2005; Meirmans and Van Tienderen, 2013; Spoelhof et al., 2017; Mason and Wendel, 2020; Blischak et al., 2023; Dunn and Sethuraman, 2024). What might be the evolutionary implications of viewing polyploidy in this way?

Box 1 **Should we abandon the binary classification of autopolyploids and allopolyploids?**

Given the conflict and confusion in the different ways polyploid categories are defined, coupled with the continuous variation that forms a gray zone, should these categories be abandoned? In practice, the terms are applied both to mode of origin (within‐ versus between‐species) and genetic properties (inheritance), which risks conflating pattern with process and how each changes through time. Proposed solutions to these terminological issues have included clarifying and standardizing use of the existing terms (Doyle and Sherman‐Broyles, 2017) and redefining categories by mode of inheritance to avoid questions around species definitions (Carputo et al., 2006); opinions on these options vary in the polyploid community (including within our authorship; see Author Viewpoints). However, any changes to the usage of these terms would be difficult, given that they are deeply entrenched in the literature. Overall, we consider that the distinction between autopolyploidy and allopolyploidy is likely to be genetically meaningful, with these terms providing coarse categories associated with the origin, form, and function of a genome, when little else may be known. Researchers must, however, be mindful of the limitations of these terms, not least in comparative studies or meta‐analyses (e.g., Barker et al., 2016), which may aggregate across studies that have defined polyploid types differently, or compare within a given polyploid type when they may have formed through a diversity of processes. Our hope is that comparative genomics of polyploid variation will provide further insights into the degree of genomic distinctiveness between conventional auto‐ and allopolyploids (e.g., see Wang et al., 2019), at which point we can make an informed decision over the future use of the terminology.

This question about defining and understanding the diversity among polyploids parallels recent work in the field of speciation genetics. Speciation has long been viewed as a continuous and gradual process (Darwin, 1859), with the term “speciation continuum” (Drès and Mallet, 2002) used to describe the continuum of distinctiveness between species, whereby some populations show stronger reproductive isolation than others, though many taxa are at various stages of incipient speciation that make them difficult to delineate. In an attempt to more rigorously define and quantify this idea, Stankowski and Ravinet (2021) defined the speciation continuum as based on measures of reproductive isolation, where a value of 1 represents complete isolation. This work once again illuminated the challenges of categorizing speciation, but also proposed a viable framework for comparative analyses (at least using this criterion for defining “species”; see de Queiroz, 2007).

Is it possible to follow this same logic to more rigorously conceptualize polyploid diversity and transition states? Our aim here is not to revise current polyploid classification (see Box 1), but to develop a framework for studying the polyploid continuum using measurable genomic features, and to illuminate evolutionary processes, subsequent to initial polyploid formation, that might generate additional biological complexity that does not readily fit into temporally static nomenclatural categories. This challenge of quantifying variation across polyploids dates back at least to Stebbins (1947), who recognized: “The decision as to whether a polyploid is auto‐ or allopolyploid must depend on the amount and type of differences that are considered significant in separating the two categories.” Here, we propose that considerations of genetic variation—sequence‐level, structural, and functional, across the genome and between related chromosomes and chromosome segments—provide a useful basis for conceptualizing the polyploid continuum (Meirmans and Van Tienderen, 2013). By focusing on the range and types of variation in cytogenetic and genomic features across the diversity of polyploids, attention may be focused on both the processes (e.g., the molecular mechanisms and evolutionary processes at play that cause transitions over time) and their outcomes (the frequency of different types of polyploids at any snapshot in time). The focus on genetic variation, a measurable criterion, sidesteps the complexity and debate associated with definitions of polyploids based on origins alone (i.e., avoiding definitions based on whether the parents of specific polyploids are different species), and accounts for variation that arises post‐polyploid formation. Just as the speciation continuum does not assume the irreversibility of the continuum, with species divergence and species collapse both possible, neither does the polyploidy continuum require fixed states, and we similarly emphasize the transition dynamics of polyploid genomic features among taxa or populations.

**Figure 1:**
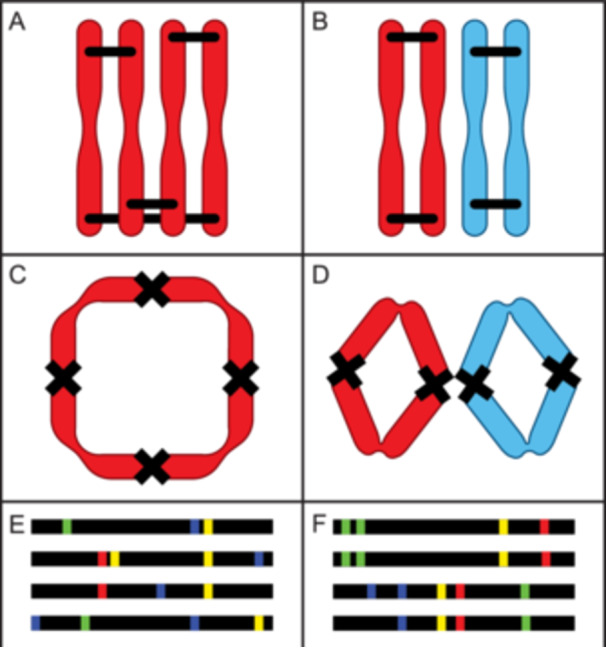
Expected outcomes at either end of a polyploid continuum. (A) Pure autopolyploids experience crossing over (black bars) between all chromosomes. (B) Allopolyploids with divergent subgenomes (chromosomes colored blue and red) have crossover events only between homologous, rather than homoeologous, chromosomes. (C) Autopolyploid experiencing polysomic inheritance, here shown with a ring formation at meiosis (see Bomblies et al., 2016, for other possibilities). (D) Allopolyploid showing regular bivalent formation and disomic inheritance. (E) Autopolyploid sequence visualization showing single nucleotide polymorphisms (colored boxes) that segregate across chromosomes (black lines). (F) Allopolyploid sequence visualization showing fixed heterozygosity between two subgenomes. Chromosome configurations in C and D are based on Gonzalo et al. (2023).

At the time of polyploid formation, polyploids produced via somatic doubling have no novel genetic variation (Figure 1A), while the hypothetical other end of the spectrum could comprise polyploid hybrids formed from crosses between two species that are divergent, have low sequence similarity, and perhaps have high structural divergence (Figure 1B). The polyploid formed via somatic doubling will initially have obligate polysomic inheritance, as the sequence‐level variation distinguishing pairs of homologs that is required for disomic inheritance is lacking (Figure 1C; for review, see Lv et al., 2024). This concept therefore anchors one end of the spectrum as “pure” autopolyploids (Spoelhof et al., 2017). At the other extreme, the polyploid formed from wide hybridization results in individuals with obligate disomic inheritance via complete preferential pairing between homologous chromosomes (Figure 1D), where there are no chromosomal interactions between distinct subgenomes. In both of these situations, there will be characteristic segregation patterns present in genomic sequencing data (Figure 1E, F), and these different modes of origin will affect the maintenance of genetic variation and its distribution into genotypes. Subsequent evolutionary change post‐polyploid formation will blur and complicate this distinction (Box 2), but these two poles provide a starting point for understanding natural diversity and the forces underlying evolutionary transitions subsequent to polyploid formation.

Box 2 **Diploidization.**

Following whole‐genome duplication, polyploids undergo a series of changes over time that make them appear more diploid‐like and obscure their polyploid ancestry (a process known as “diploidization” or “rediploidization”). In classic autopolyploids, this may involve a transition from polysomic inheritance, to the derivation and maintenance of disomic inheritance (sometimes called “cytological diploidization”; Li et al., 2021). Once disomic inheritance is established, there are then a cascade of possible subsequent genomic changes, including sequence divergence, a reduction of chromosome number, and the loss of duplicate gene copies (sometimes called “genic diploidization” or “fractionation”; Li et al., 2021; Gundappa et al., 2022). Diploidization has a significant bearing on polyploid classification, as a “diploidized autopolyploid” may have divergent duplicate chromosomes or chromosome segments that are indistinguishable from those in a classic allopolyploid. This scenario leads to the surprising possibility that “apparent allopolyploids” (i.e., those that exhibit duplicated chromosomes with exclusive and preferential bivalent pairing) may hypothetically be derived from lineages that had an autopolyploid origin. The genomic changes following diploidization may also, in theory, populate much of the space across the polyploid continuum, and underlie major genome‐wide changes and evolutionary transitions (discussed in this review). Despite its importance, little is known about the mode and tempo of diploidization across plants.

## QUANTIFYING GENOMIC DIVERSITY ACROSS THE POLYPLOID CONTINUUM

### Genome‐wide heterozygosity

Heterozygosity is a widely used measure of genetic variation in population genetic studies of polyploids (Meirmans et al., 2018). Heterozygosity plays a critical role in the evolutionary outcomes of polyploids, with Stebbins (1985) proposing that polyploid success is “almost always the result of increased heterozygosity,” which can result in phenotypic heterosis (Washburn and Birchler, 2014). Heterozygosity in polyploids is a product of “fixed heterozygosity” that arises either as divergence between homoeologs from different subgenomes in classic allopolyploids, or as whole‐genome‐derived duplicates in “diploidized autopolyploids” that exhibit preferential and exclusive bivalent pairing but have an autopolyploid origin (Box 2); and in “segregating heterozygosity” (diversity among recombining homologs) (Soltis and Soltis, 2000). Fixed heterozygosity is a hallmark of disomic segregation, produced by separate homoeologous subgenomes in classic allopolyploids or from divergence between WGD‐derived duplicates in “diploidized autopolyploids,” each of which has independent segregation of alleles. In contrast, segregating heterozygosity among recombining homologs is expected under polysomic segregation (or the result of homoeologous exchanges), produced by four (in a tetraploid) homologous chromosomes that pair randomly during meiosis and hence segregate independently. As such, heterozygosity provides a link between mode of inheritance, which cannot currently be measured at scale across diverse wild polyploid systems (instead requiring segregating populations), and modern genomic data sets. Furthermore, measures of heterozygosity can be universally applied across polyploids, unlike some other measures used to quantify divergence (discussed below), which cannot be applied to traditionally conceived autopolyploids that do not possess subgenomes.

Approaches that quantify the amount and reveal the genomic distribution of fixed heterozygosity are informative in this respect (e.g., Scott et al., 2023). Such approaches also have relevance for understanding polyploid origins and subsequent evolution. The presence of fixed heterozygosity from interspecific hybridization may lead to phenotypic heterosis at the point of initial formation—due, for example, to masking of the effects of subgenome‐specific deleterious alleles or to heterotic combinations of favorable allelic combinations (Soltis and Soltis, 2000; Chen, 2010). One might speculate that fixed heterozygosity may be important for initial polyploid establishment or niche differentiation from progenitor diploids. In polyploids exhibiting polysomic inheritance, allelic copies are more similar to each other, on average, at the point of formation than in polyploids with disomic inheritance. Genetic variation (heterozygosity) transferred via unreduced gametes into novel polysomic polyploids is then subject to natural selection, unlike fixed heterozygosity, where homozygosity within and separation between each subgenome means there is no segregating variation for selection to act upon.

Thus, the forms and amounts of heterozygosity matter with respect to post‐polyploidization evolutionary trajectories. There are, however, notable challenges in quantifying heterozygosity across the polyploid continuum. For example, population genetic studies of polyploids with easily distinguishable subgenomes that use “gold standard” chromosome‐level reference genomes will tend to only estimate diversity within rather than between subgenomes, because the constituent subgenomes are separated in most reference genomes, even without the use of haplotype phasing approaches. Clearly, future methodological development in this area will be welcome, with polyploid‐specific pangenome analyses (e.g., Bird et al., 2025) proving especially useful in quantifying the different forms of heterozygosity in polyploids and its genomic distribution.

### Genome‐wide sequence divergence

The extent of sequence similarity or divergence between regions derived from whole‐genome duplication, including homoeologs in classic allopolyploids, represents another metric that can be used to quantify polyploid diversity, as a natural extension of the considerations of heterozygosity described above. An advantage of this metric over genome‐wide heterozygosity is that it can be derived from a single high‐quality genome assembly. Therefore, this measure can be calculated without population‐level sampling, though such sampling will provide a more general estimate representative of the population or the species. However, a limitation of this approach is that it cannot be applied to conventional autopolyploids, which do not possess divergent homoeologs.

Direct sequence comparisons from chromosome‐level genome assemblies have been performed in model organisms such as species in the genus *Arabidopsis*. For example, the genome of allopolyploid *Arabidopsis suecica* shows only 86% sequence identity based on whole‐genome alignments performed between the subgenomes, reflecting a high degree of sequence‐level divergence (Burns et al., 2021). However, most polyploid genomic studies do not report this metric, even though it is tractable to estimate across shared duplicated regions genome‐wide. One complication with this calculation is that it must consider sequence divergence and copy‐number changes (e.g., TE‐associated insertions/deletions) that can render some regions effectively hemizygous in intergenomic comparisons. Other approaches for measuring homoeolog divergence (i.e., subgenome divergence in a conventional allopolyploid) include estimates made directly from short sequences (*k*‐mers) generated from unassembled sequence reads, avoiding the complexity of polyploid genome assembly. Here, mathematical models are fit to the *k*‐mer spectrum, with a higher 2*x* peak (in a tetraploid) indicating greater subgenome divergence (Becher et al., 2020). However, these approaches are limited by the information content of short sequences and the constraints of these mathematical models (e.g., currently only being applicable to tetraploids). Pangenome graphing methods (e.g., Bayer et al., 2021; Garrison et al., 2024; MacNish et al., 2025), haplotype‐based sequencing (e.g., Snyder et al., 2015), and advances in karyotype reconstruction (Lysak et al., 2016) will provide better estimates of sequence‐level divergence of polyploids in the future.

### Gene‐level divergence

A more established way to estimate the divergence between homologous and homoeologous chromosomes is based on measurements using gene sequence data rather than genome‐wide inference. This approach avoids the complexities of including and comparing less‐conserved, repetitive non‐genic regions, with the caveat of overlooking structural variation that might influence pairing (discussed below). One example where gene‐level divergence has proved informative is cotton (*Gossypium*), in which species differ nearly twofold in (sub)genome size, but differ by only ~2.7% in synonymous substitutions (*d*_S_) in the genic fraction (Page et al., 2013; Chen et al., 2020); thus, only ~175 kilobases at the terminus of one chromosome has experienced a homoeologous exchange event (Conover et al., 2024), among the lowest published estimates of any polyploid with easily distinguishable subgenomes. This approach of measuring divergence in genic regions has been scaled to many taxa, with a recent example using six independent polyploid species for thousands of nuclear loci (Sharbrough et al., 2022), where estimates varied markedly between species, from average *d*_S_ = 0.026 in Arabica coffee (*Coffea arabica*) to *d*_S_ = 0.105 in quinoa (*Chenopodium quinoa*). Notably, in the analysis of these genomic data sets (and often in other similar studies), precautions were taken to ensure that genes used in these estimates fit the “quartet” tree topology expected in polyploids that form via hybridization between extant diploid progenitors, which, if not accounted for, would artificially reduce the expected divergence between homoeologous gene copies. Overall, estimating average divergence from a suite of informative genes is likely to be an easy‐to‐implement approach for measuring diversity in a large number of polyploids, although it will not convey the full spectrum of polyploid variation (discussed below).

### Structural variation

Structural genomic variation that distinguishes homologous or homoeologous chromosomes is also an important measure, which will reflect both initial conditions at polyploid formation, and post‐polyploid formation processes (see Box 2). Measures of structural variation, such as the frequency and length distribution of structural variants, are increasingly accessible from polyploid genome data (Healey et al., 2024). It is unclear, however, how predictive these descriptions or summaries of structural genomic variation might be with respect to chromosomal behavior and segregation patterns in polyploids. Structural variants may have a significant impact on chromosome pairing (reviewed by Lv et al., 2024), phenotypic traits (reviewed by Schiessl et al., 2019), and genome evolution trajectories (reviewed by Mason and Wendel, 2020). Although structural variants that differ between chromosomes are not the only or even the primary reason for disomic inheritance (Nyarko and Mason, 2022; Bomblies, 2023), in polyploids with low sequence‐level divergence between sets of homologous chromosomes, structural differentiation is a probable way that polysomic inheritance can change to disomic inheritance over time. Indeed, there have been observations of predominantly polysomic polyploids where chromosomal rearrangements involving one or two chromosomes have resulted in disomic inheritance of these chromosomes, giving rise to a mixed inheritance type (reviewed by Lv et al., 2024). This exemplifies, at the level of individual chromosomes, a theme of the present review: that observed genomic features that are often used to characterize polyploid “states” may be fluid and temporally dynamic, changing over time.

## EVALUATING THE UTILITY OF THE POLYPLOID CONTINUUM CONCEPT

Can individual genetic measures (such as those discussed above) act as metrics to describe variation among polyploids, in a manner similar to reproductive isolation in the speciation continuum? And how will studies of genomic variation across a broad spectrum of polyploids map to or inform the simplistic binary distinction between auto‐ and allopolyploids? Insights into these questions are likely to come with the increasing availability of high‐quality haplotype‐phased polyploid genomes, coupled with comparative genomic analyses and population genomics inference tools. Such analyses will, hopefully, elucidate relationships among specific features of genomic divergence, and the characteristics and fates of derived polyploids. For example, comparisons of various measures of gene content differences and structural divergence across taxa may lead to deeper insights into how the origin and derivation of particular polyploids is related to the various metrics described above.

**Figure 2:**
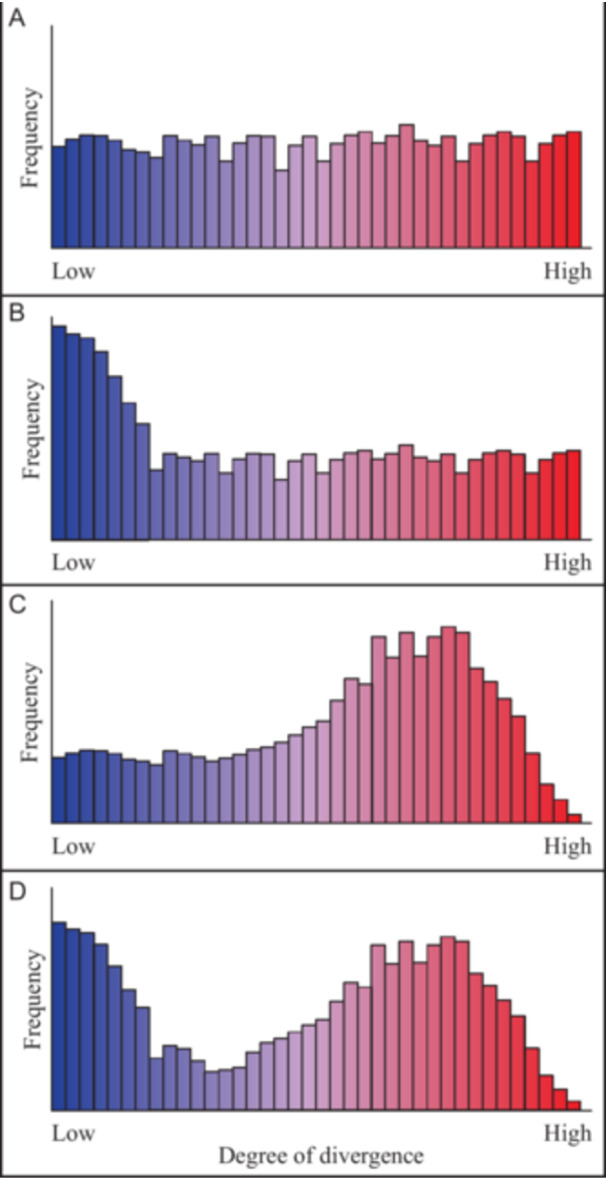
Selected models showing the possible frequencies of different types of polyploid taxa. Each plot shows how a measure of polyploid diversity (such as fixed heterozygosity) may be predicted to vary across the diversity of polyploid plant species. (A) Simple model, where there is no general trend in divergence between homologous or homoeologous chromosomes across species. (B) Autopolyploidy‐predominant model, where the relatively high abundance of (potentially ephemeral) autopolyploids leaves a distinct signal. (C) Bounded‐formation model (“sweet spot model”), reflecting a typical degree of parental divergence in allopolyploids and the scarcity of species with highly divergent subgenomes. (D) Combined model, including the relative abundance of autopolyploids and the typical degree of parental divergence in allopolyploids, showing a characteristic dip at moderate frequencies of divergence. These models illustrate a range of possible scenarios that could be tested with genomic data. Colors indicate the spectrum of variation, between the classically recognized alternative end points of autopolyploidy (blue) and allopolyploidy (red).

As one simple model of variation across polyploid taxa, we might expect no general trend with respect to any single measure of sequence or structural variation as discussed above (Figure 2A). This would demonstrate the highly variable nature of polyploid genomic diversity and/or the poor explanatory power of any single measure of genetic variation. It may be, however, that the occurrence of recently formed, and potentially ephemeral, autopolyploids (Lewis, 1980; Ramsey and Schemske, 1998; Salony et al., 2024) distorts the frequency spectrum of different polyploids such that it is left‐skewed (Figure 2B). Alternatively, or in addition, it is widely anticipated that polyploids with more divergent subgenomes will have higher fertility, up to a point, as chromosomes will more regularly form bivalents in meiosis (Bomblies, 2023), thereby increasing their fitness and, thus, their chance of persisting in the long term. As a counterbalancing evolutionary dynamic, however, divergent species are less likely to hybridize in the first place (Brown et al., 2023), and as such there may be a “sweet spot” degree of divergence among diploids that makes polyploidization more likely to be successful (Rothfels, 2021), with species being close enough to hybridize, but distant enough to enforce regular, disomic chromosomal inheritance. This may be reflected in the frequency of different classes across the ploidy continuum (Figure 2C, and represented as one peak in Figure 2D). Notably, Buggs et al., (2009) found that polyploid formation occurs between species with a wide range of observed sequence divergence, suggesting that much of the polyploid continuum may be populated, at least at the time of formation. Further genome‐based data collection across independently derived instances of polyploidy will make it possible to evaluate and refine these models, and to test whether there are general trends across species or the outcomes of polyploidy are lineage‐specific.

Although comparative approaches such as those described above will be informative, at present it remains unclear which aspects of sequence or structural divergence are the most important axes of variation—and how relationships among these aspects vary across plant lineages—in terms of chromosome behavior, genetic segregation, and the future fate of a polyploid. It may be predicted that chromosome size differences, and the genomic variation they reflect, might play a more important role than, say, genic divergence or point mutations in influencing chromosome behavior. For example, homoeologous exchanges are quite rare in *Gossypium* (Conover et al., 2024), in which the chromosomes of one subgenome (and its progenitor diploid) are twice as large as in the other subgenome (and the other progenitor diploid), while homoeologous exchanges are common in many other allopolyploids with lesser degrees of subgenome size difference (Samans et al., 2017; Mason and Wendel, 2020). However, chromosomal behavior is clearly not influenced by chromosome size alone, as there are notable cases of syntenic chromosomes with similar subgenome sizes but almost no homoeologous recombination (e.g., bread wheat, where homoeologous recombination is prevented by the *Ph1* locus; Griffiths et al., 2006). Overall, while sequence divergence at the genic level and number of structural variants may prove interrelated in some cases, comparative analyses of diploid genomes from 32 plant genera showed high variability in the rate at which chromosomal inversions are fixed, with the number of inversions uncorrelated with sequence divergence (Hirabayashi and Owens, 2023). Both sequence and structural variation may be expected to accumulate with time since polyploid formation, though (as in diploids) the relationship between these two measures may not be linear. Moreover, not all structural variants will be equivalent with respect to evolutionary outcomes; it is possible, for example, that large structural variants are more important than small indels in repeats, but how they compare in evolutionary impact to each other and to genic divergence is unknown. Also, divergence in sequence and localized synteny are frequent enough to accumulate relatively linearly over time, while larger structural changes such as inversions and translocations occur more episodically and will have a greater impact in terms of synteny (Grant, 1981; Mandáková and Lysak, 2018). In this respect, both genic mutations and structural changes that affect chromosome pairing in polyploids may shape longer‐term evolutionary outcomes.

Overall, it is clear that the assumptions about the genomic distinctiveness of classic auto‐ and allopolyploids are overly simplistic. Accordingly, it is evident that no simple, single proxy can capture the full spectrum of variation across the polyploid continuum, even if such a proxy provides a useful summary of variation and helps frame future research (discussed below). At the point of formation, some separation may exist between conventional auto‐ and allopolyploids, especially with respect to inheritance: allopolyploids typically rely on mechanisms that discriminate between homologous and homoeologous chromosomes, whereas autopolyploids achieve segregation via control of crossover frequency and location (reviewed in Bomblies, 2023). Intermediates between disomic and polysomic inheritance appear to be uncommon (but see Stift et al., 2008). Over evolutionary time, however, additional layers of complexity may blur this initial separation, in particular due to post‐formation processes that either increase or decrease the extent of genomic differences between homologs and homoeologs (see below), and these must be considered when evaluating models of polyploid diversity.

## DEVELOPING A MORE REALISTIC LANDSCAPE OF POLYPLOID VARIATION

**Figure 3:**
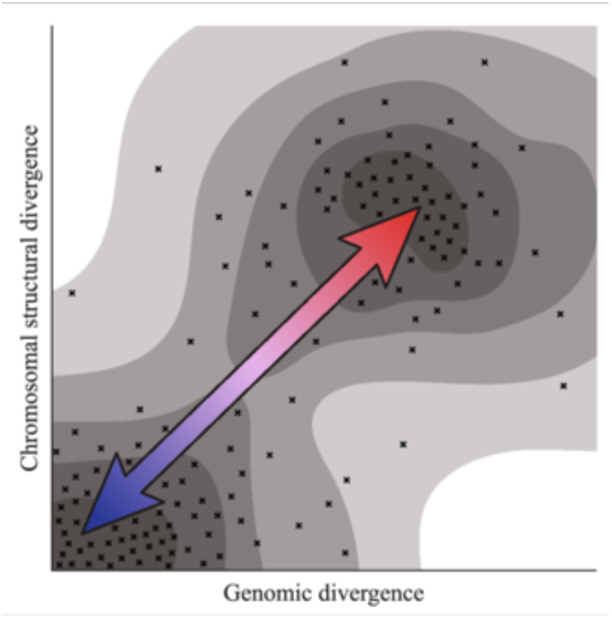
A hypothetical hyperspace of possible genotypes across polyploid diversity, shown as a simplified two‐dimensional representation. Polyploids vary in multiple aspects of their genome structure, here with two hypothetical factors on the axes: a measure of genomic divergence (such as *K*_s_ or percent sequence divergence) and chromosomal structural divergence (such as total length of shared synteny blocks between haplotypes). Genotypes (crosses) are most likely to occupy certain parts of poly‐space (darker gray background). Species are not fixed in their position, with transitions between the classically recognized alternative end points of autopolyploidy (blue) and allopolyploidy (red).

A more elaborated view of the polyploid continuum than that presented above (Figure 2) would be in multidimensional space, as a multivariate projection integrating many variables relevant to polyploid outcomes (Figure 3). This idea builds on the discussion above, recognizing that any given polyploid species does not necessarily occupy a single fixed point on a simple linear continuum, but that individuals from different populations within a species may vary (Box 3; Paape et al., 2018), that duplicated sequences may diverge (Chen et al., 2020) or become more similar (Mason and Wendel, 2020; Wu et al., 2021; Han et al., 2025) over time, and that different measures of sequence and structural divergence may not be perfectly correlated (Hirabayashi and Owens, 2023).

Box 3 **Population genomics of polyploidy: dynamics and evolutionary consequences.**

New understanding of polyploid genome dynamism and the selection and demographic pressures shaping polyploid genomes is increasingly emerging not just from single genome studies, but from population genomic research. Numerous studies show the importance of standing genetic variation on polyploid evolutionary outcomes, highlighting the value of studying variation across diploid progenitors and the resulting polyploid progeny, which is increasingly feasible with pangenomes (Gordon et al., 2020; Hämälä et al., 2024). Such work is needed to reveal the pace of change soon after polyploid formation, and the extent to which this generates diversity along the polyploid continuum. As the number of studies grows, we expect increasing insights into the diversity of processes operating across polyploids of different origins, and insights into the extent at which these processes are controlled by neutral or selective forces (Meyers and Levin, 2006; Clo et al., 2022). An illustrative example comes from population studies of polyploid *Arabidopsis* at the two extremes of the polyploid continuum, where there were minimal differences in purifying selection between tetraploid and diploid populations of *Arabidopsis suecica* (Monnahan et al., 2019), but the subgenomes of tetraploid *A. kamchatica* (formed through the hybridization of *A. lyrata* and *A. halleri*) exhibited fewer deleterious mutations and more neutral to slightly deleterious mutations compared to its diploid counterparts (Paape et al., 2018). While these analyses might suggest that the difference in inheritance patterns between these two polyploids (polysomic in *A. suecica* and disomic in *A. kamchatica*) could cause a difference in the efficacy or mechanism of purifying selection, it may also be an idiosyncratic outcome observed in these specific species (Monnahan et al., 2019; Conover and Wendel, 2022; Hämälä et al., 2024). In *Gossypium*, for example, deleterious mutations were shown to accumulate faster in polyploids than in diploids (Conover and Wendel, 2022), with the rate of accumulation also differing between the two subgenomes of polyploids. Additionally, methodological complications arise when comparing polyploids with disomic and polysomic inheritances. Because the subgenomes of classic allopolyploids are often analyzed independently and treated as if they were separate diploid lineages that coincidentally reside in the same nucleus, these analyses will not capture the epistatic interactions of alleles co‐segregating in the two subgenomes. Polyploids with polysomic inheritance, however, cannot be analyzed in a similar diploid framework. Thus, confidently attributing any of these patterns to differences in how natural selection operates depending on mode of formation, or as a consequence of polyploidy per se (rather than resulting from the numerous other factors such as population size changes, transitions to self‐compatibility, or recombination rates, to name a few) remains a methodological challenge for studying polyploids on a population level. Future modeling work may assist in developing population genetics frameworks and inference tools that can model the full interactions between alleles regardless of segregation pattern. This includes modeling the allelic dominance patterns acting on the four alleles in tetraploids with polysomic inheritance and the “homoeologous epistatic dominance” (Conover and Wendel, 2022) acting between the two independently segregating paralogs in polyploids with disomic inheritance. Such developments could build on recently developed models (e.g., Blischak et al., 2023; Dunn and Sethuraman, 2024) to account for how natural selection might operate differently on varying ploidal levels, and along the continuum of subgenome divergences.

What might be the axes in the higher‐dimensional graph that capture this variation? This question may best be answered using comparative genomic analyses of a range of species across polyploid diversity, representing diverse clades and ecological settings. The axes of variation could be explored initially using a dimensionality‐reduction approach such as principal component analysis, allowing for an evaluation of the loading of different factors or attributes on each axis. Obvious candidate factors that may vary across polyploids would include the measures of diversity and divergence discussed above, as well as other features such as transposable element (TE) distribution and relative synteny (Meca et al., 2024). Such a visualization may reveal hotspots of biological realism (in terms of highly occupied parts of the “poly‐space”), while other areas of poly‐space are sparsely occupied or empty. Conceptually, this thinking could extend to theoretical explorations of expected clustering of polyploids under different evolutionary processes.

One benefit of projecting polyploidy across a multidimensional landscape is how this representation may better capture the temporally dynamic nature of polyploid genomes and act as a framework for understanding evolutionary transitions. As an example of this temporal dimension of the issue, it typically will be unclear whether any single measure, such as sequence divergence between homologous or homoeologous sequences, represents the initial conditions found at the time of polyploid origin, or if, instead, distinctions and similarities reflect derived conditions that have evolved since polyploid formation. For example, a genome with divergent duplicate sequences could originate via allopolyploidy, as assumed from initial observations, or instead from a now‐cryptic autopolyploidy event followed by subsequent divergence of duplicate gene copies. Here, divergence will have accumulated over time to a degree that a naive observer might reject an autopolyploid origin in place of an allopolyploid origin with subsequent extinction of the diploid progenitors. Additionally, polyploids that arise through the hybridization of different species may lack sufficient sequence differentiation to ensure complete preferential pairing of chromosomes, ultimately resulting in a polyploid that appears to have all the characteristics of an autopolyploid origin. This pathway was recently shown in polyploid rice, synthesized from diploid parents representing subspecies *japonica* and *indica*. The polyploid experienced subsequent genome‐wide recombination (and/or partial preferential pairing) between parental chromosomes, such that after four to twelve generations of selfing, a wide spectrum of homoeologous chromosomal recombination products was generated (Wu et al., 2021; Han et al., 2025). In this case, the end result was different lines that contain a mosaic of chromosomal regions with variable dosages from each diploid parent, and in nearly all imaginable combinations, including the complete removal or fixation of regions from one or the other progenitor genome (as modeled in Mason and Wendel, 2020). Moreover, the spectrum of resulting progeny varied in selectively relevant phenotypic traits, including flowering time and yield (Wu et al., 2021; Han et al., 2025). Diverse chromosomal variation is also present in natural populations of polyploid species, with, for example, wild populations of neoallopolyploid *Tragopogon miscellus* showing diverse intergenomic translocations indicating prolonged chromosomal instability (Chester et al., 2012), and many established crops (e.g., rapeseed and peanut) showing non‐homologous recombination often associated with phenotypic diversity (reviewed by Schiessl et al., 2019).

Another illustrative example of the complex evolutionary outcomes of polyploidy concerns the classic concept of segmental allopolyploidy (Stebbins, 1947; Mason and Wendel, 2020). This category recognizes polyploid genomes that contain sets of chromosomes varying in their degree of cytological homology with each other, such that segmental allopolyploids represent an intermediate between classic auto‐ and allopolyploidy (e.g., the peanut genome; Bertioli et al., 2019). Segmental allopolyploids were conceived as a category to explain multivalent chromosome‐pairing behavior in polyploids resulting from interspecific hybridization, which is now understood to instead reflect a diversity of factors and processes (reviewed by Bomblies, 2023). However, a mix of polysomic and disomic inheritance due to partial preferential pairing has been observed in a number of polyploid species (reviewed by Lv et al., 2024) and could arise via multiple distinct mechanisms. First, they could emerge via homoeologous exchanges in polyploids formed with initially strong subgenome differentiation, where a segment from one subgenome is replaced by the other, rendering that segment identical across all chromosomes (Mason and Wendel, 2020; Deb et al., 2023; Han et al., 2025). Second, in polyploids with polysomic inheritance, the introduction of novel structural rearrangements that differentiate chromosome copies may result in partial preferential pairing of homologs that share similar structural rearrangements, leading to a mix of polysomic and disomic inheritance (Lv et al., 2024). Finally, hybridization between parental species that lack fully differentiated genomes, such as where there have been blocks of introgression between parental progenitors, may be anticipated to have a mix of modes of inheritance. As demonstrated by these examples, similar chromosomal/cytogenetic phenotypes may arise from different processes and dynamics. Whether a more complex landscape of poly‐space can reliably distinguish such different dynamics leading to similar outcomes is unclear; however, this seems to be a promising route of investigation, especially if it could capture aspects of genomic heterogeneity that may differ depending on the underlying process.

A further consideration is whether this polyploid landscape can be extended to other ploidal levels, including classically recognized diploids that we now understand have a paleopolyploid ancestry. Ancient polyploidy events characterize all angiosperm lineages, such that “today's diploids are yesterday's polyploids” (Wolfe, 2001; Jiao et al., 2011). While genomic changes following diploidization are likely to occur differently depending on the extent of differentiation between duplicate gene copies at the time of polyploid formation, the delineation between a polyploid and the diploid it may eventually become can be difficult, if not impossible, to identify. For example, many newly formed polyploids behave like diploids in having disomic segregation, and thus have segregation patterns similar to those of diploids but with a doubled complement of chromosomes and genes (Wendel, 2015). Thus, the primary distinction between these polyploids with disomic inheritance and their diploid counterparts is the number of chromosomes and genes in the genome; however, because the number of genes never returns to exactly the number that existed in the pre‐polyploid ancestor, there is no obvious or currently measurable end point of when a polyploid becomes a diploid. Thus, while the saltational transition from diploidy to polyploidy allows for simple categorization between ploidal levels, the same cannot be done for the protracted, gradual transition from polyploidy to diploidy. As such, readily separating diploids from polyploids at any static time point may have as many caveats and complications as separating autopolyploids from allopolyploids. In developing an empirical understanding of polyploid genomic diversity, we should additionally consider these transition events, as well as the historical hallmark of older polyploidy events in relation to subsequent cytological diploidization and genome evolutionary dynamics (Li et al., 2021).

In this respect, comparative genomics may also prove useful, if data are generated from lineages with both contemporary polyploidy and historical polyploids of varying ages, for understanding the mode and tempo of diploidization (Li et al., 2021) and disentangling lineage‐specific effects from more general patterns. For example, polyploids may experience different speeds at which polysomic inheritance shifts to disomic inheritance (Li et al., 2021). At one extreme, some chromosomes in teleost fishes have retained tetrasomic inheritance patterns for more than 60 million years (Parey, 2022), while at the other, the holocentric chromosomes of sedges are thought to have facilitated multiple end‐to‐end chromosomal fusion events, causing an octoploid to evolve disomic inheritance and extensive chromosome number reduction <4 million years after formation (Hofstatter et al., 2022). Such comparative studies across lineages may be able to disentangle whether there are additional genomic signals related to the speed of diploidization that should be incorporated into an expanded polyploid continuum framework. This work would build on well‐established findings, such as those demonstrating that historical polyploidy events can often be detected on the basis of a diagnostic peak in the distribution of *K*_s_ values for sampled paralogous gene pairs (Cui et al., 2006; McKibben et al., 2024), and that historical polyploidy can be dated by using the age of shared TE insertions across polyploid subgenomes (Shen et al., 2023)—with the note that these methods may actually be dating characteristics of the subgenomes, and not the time of polyploid formation per se (Thomas et al., 2017). Such genomic features exemplify the kinds of additional axes in a visualization of the relevant poly‐space (Figure 3).

Extending the polyploid continuum to include higher polyploids may also prove informative with respect to understanding evolutionary process, notwithstanding the experimental challenges inherent in this undertaking. Plants vary enormously in their ploidal levels, with the highest chromosome number of any plant being 2*n* = 1440 in *Ophioglossum reticulatum*, which may reflect 48‐fold or 96‐fold ploidy (depending on the assumed base chromosome number; Khandelwal, 1990). High polyploids introduce additional complexity, due to technical issues with genome assembly and comparative analyses in addition to inherent biological complexity. Despite these challenges, progress has recently been made in the genomic analysis of complex polyploids. For example, a recent study resolved a haplotype‐level genome assembly for the large (8 Gb) genome of the invasive Japanese knotweed (*Reynoutria japonica*), which proves to be an octoploid (2*n* = 8*x* = 88) with a mixed auto‐ and allopolyploid history (i.e., autoallopolyploid; Kostoff, 1939) with genome constituents AAAABBBB (Wang et al., 2025). Contextualizing such taxa on a polyploid continuum is challenging, requiring consideration of complex genome dynamics, but we expect insights to be facilitated by improved genomic resources and methodological advances.

### Toward a synthesis

Viewing polyploid variation as a continuum or complex landscape, rather than through the traditional binary lens, offers a fresh perspective that can be brought to bear on understanding the evolutionary dynamics shaping the spectrum of polyploid diversity. This viewpoint may encourage researchers to look for general principles and explanatory processes in a field that has often looked for differences. For example, hybridization is a common feature shaping the evolution of all polyploids (Harlan and de Wet, 1975), not just allopolyploids, with hybridization occurring across ploidal levels (reviewed in Bartolić et al., 2024, and Brown et al., 2024), and as a key factor in the generation of diversity in many species‐rich, taxonomically complex polyploid groups (Ennos et al., 2005). Moreover, this approach may allow us to revisit classic questions in polyploid research in a new light, providing a framework for the integration of new data sets (see Soltis et al., 2010; Fox et al., 2020). For example, a major area of research is in the investigation of the diverse evolutionary consequences of polyploidy, from the genomic changes that affect the transcriptome, proteome, and metabolome to how polyploidy affects morphology, ecology, and reproductive success. Many comparative reviews have contrasted these aspects between diploids and tetraploids, and between auto‐ and allopolyploids (e.g., Levin, 1983; Comai, 2005; Chen et al., 2007; Ramsey and Ramsey, 2014). Moving from comparisons in this binary framework to more open comparisons of the relationship between polyploid genomic diversity and other biological traits may help in the search for general outcomes of polyploidy. In this respect, scattered but growing evidence suggests that the genomic variation present across polyploids correlates with, and may cause, a range of important biological outcomes. For example, the extent of subgenome divergence may, in turn, impact genome‐wide expression patterns (reviewed in Edger et al., 2018). However, even with more data, any search for general rules governing polyploid subgenome evolution may remain elusive due to idiosyncratic outcomes in different taxa. Accordingly, it is likely that with respect to many phenomena, general conclusions will likely represent modal behaviors rather than rules, with idiosyncratic lineage‐specific effects and evolutionary vicissitudes overlying more general outcomes.

## CONCLUSIONS

Appreciating the complexity of nature is an endlessly fascinating and challenging task, with the categorization of polyploids being a topic that has been frequently considered but is far from being resolved. The terms “autopolyploidy” and “allopolyploidy” are both useful and deeply embedded in the literature on polyploid origins and evolution, but classical conceptions of these terms may constrain a fuller appreciation of biological possibilities and dynamism because of preconceived notions or assumptions. They retain utility as descriptors of polyploids at any one snapshot in time, but it is important to recognize, as others have previously, that these two terms represent hard‐to‐delimit points on a continuum or multidimensional landscape that has multiple underlying biological underpinnings and definitions, is temporally dynamic, and is taxonomically as well as genetically highly variable. We expect that the further application of high‐quality genome assemblies across the phylogenetic spectrum will teach us much about polyploid diversity and its complex and intricate dynamics.

## AUTHOR VIEWPOINTS

This paper reflects our collective insights into the biological complexity of polyploid diversity. Here, we describe why each of us wanted to contribute to this review, and our own individual opinions on polyploid diversity and classification.

**Alex D. Twyford:** My interest in polyploid classification started when we began assembling polyploid genomes that did not sit neatly in either the autopolyploid or allopolyploid box. An inspiring sabbatical with P.S.S., D.E.S., and J.F.W. led to us drafting a paper on the polyploid continuum, which grew into this broader review paper with valuable input from coauthors with diverse viewpoints from across the polyploid community. Writing it deepened my appreciation of polyploid variation at formation and in the derived state—it amazes me how seemingly similar outcomes can occur via such different processes. I still think the widely used polyploid terminology is useful for the crude classification of species, and that it is predictive of general genomic features, while the polyploid continuum concept is a more flexible way to frame evolutionary research studying diverse polyploids.

**Justin L. Conover:** Some of my previous work developed phylogenetic and population genetic frameworks for understanding how selection and demography shape genetic diversity in polyploids compared to diploids. In this work, and while writing this manuscript, I realized that conflicting definitions of auto‐ and allopolyploids (based on inheritance or formation) create artificial semantic distinctions lacking strong biological basis, similar to issues in describing “diploidization.” I believe that inheritance type is a key, measurable axis of variation within polyploids, and that evolutionary inferences about polyploid formation are simply hypotheses that can only be rejected if the proper rigorous frameworks and tools exist to differentiate between competing hypotheses. Ultimately, I view the auto/allo distinction as overly simplistic and, at worst, a semantic barrier to truly and deeply understanding polyploid biodiversity.

**Jeff J. Doyle:** In my experience, taxonomy does not align well with biological reality. That, together with having participated in the interminable debate over “species,” explains my skepticism about the usefulness of the traditional binary definition of polyploids. Genomic data are confirming how the massively homoplasious phenomenon of polyploidy can produce taxa that deal with the challenges of a doubled genome in many different ways and at differing tempos. I was invited to join this project after reviewing the earliest version of the manuscript, which I found unsatisfyingly neutral about the shortcomings of the binary categories. Working with the other authors to achieve a consensus for this paper, I've broadened my thinking—and retained my doubts.

**Annaliese S. Mason:** I think inheritance type (polysomic, disomic, or apomictic) is an important defining characteristic of polyploids, despite evidence for intermediate inheritance in some taxa, possible shifts in inheritance type over time, and the direct dependence of inheritance type on other characteristics such as sequence similarity between subgenomes. I think differentiating between polyploids which arise from one vs. more species is also helpful if we define species as the ability to interbreed and produce fertile(ish) progeny, and can predict likely polyploid outcomes. However, combining all polyploid characteristics to describe a continuum may better explain polyploid establishment, persistence, and evolutionary trajectories over time.

**Douglas E. Soltis:** I have been fortunate to study polyploidy for a while now—across the evolution of multiple methodologies and across the tree of life. It is human nature to categorize, but the complexities of polyploidy were ingrained in me as a student. My interests in phylogenetics, genomics, and population genetics have made it fun to view polyploid evolution as a three‐dimensional complex space, through time, varying across the tree of life. Every polyploid tells a story through time. I am excited for the future, when we will have a better understanding of both polyploid genetics/genomics and lineage (tree of life) effects in polyploid evolution. It is always a privilege to learn from others “puzzled by polyploidy,” develop new perspectives, and hopefully provide one (or at least 0.5) new idea.

**Pamela S. Soltis:** I have long used the terms “autopolyploid” and “allopolyploid” and have long been dissatisfied with the simplicity of the various definitions because the criteria don't necessarily align. Polyploidy is complex. “Segmental allopolyploidy”—“intermediate” between auto‐ and allopolyploidy—fails to clarify the diversity of polyploids, given that a polyploid that is “intermediate” could have incomplete chromosome pairing because the parental species lack fully differentiated chromosomes or because it is a classic “autopolyploid” in the process of evolving pairing. I think the essence of the problem is that nearly all properties of polyploids can be obtained by multiple processes. Only a multivariate landscape that includes time can begin to represent the beautiful diversity of polyploids. I thank my colleagues for the discussions that have led to this current exploration!

**Jonathan F. Wendel:** For me, the realization that this paper was timely emerged from the many recent advances in comparative genomics as applied to both artificial and synthetic polyploids in plants. These advances were transforming the way I thought about polyploidy and led me to consider the temporal dynamics of natural polyploid systems. In particular, I became fascinated by the possible transitions, at various evolutionary timescales, between “categories” of polyploidy that too often are presented in textbooks and many current papers as “types,” without consideration of the possibly ephemeral nature of these states. My hope is that this paper stimulates additional thinking and research in this area.

## AUTHOR CONTRIBUTIONS

All authors contributed to writing the manuscript and approved the final submission.
